# A rapid review and narrative synthesis of the evidence for oral sodium chloride supplements in the management of heart failure

**DOI:** 10.1093/ehjopen/oeag017

**Published:** 2026-02-06

**Authors:** Joseph J Cuthbert, Dionisius R Ardiyanto, Omar Amin, Sarah Greenley, Thirimon Moe-Byrne, Andrea Hilton, Lee Middleton, Jon Bishop, Laurence Humphries-Davies, Miriam J Johnson, John G F Cleland, Andrew L Clark, Maureen Twiddy

**Affiliations:** Hull York Medical School, University of Hull, Cottingham Road, Kingston upon Hull HU6 7RX, UK; Hull University Teaching Hospitals NHS Trust, Castle Hill Hospital, Castle Road, Cottingham, Kingston upon Hull HU16 5JQ, UK; Hull York Medical School, University of Hull, Cottingham Road, Kingston upon Hull HU6 7RX, UK; Hull York Medical School, University of Hull, Cottingham Road, Kingston upon Hull HU6 7RX, UK; Hull York Medical School, University of Hull, Cottingham Road, Kingston upon Hull HU6 7RX, UK; Hull York Medical School, University of Hull, Cottingham Road, Kingston upon Hull HU6 7RX, UK; Hull York Medical School, University of Hull, Cottingham Road, Kingston upon Hull HU6 7RX, UK; University of Birmingham, Edgbaston, Birmingham B15 2TT, UK; University of Birmingham, Edgbaston, Birmingham B15 2TT, UK; Person with Heart Failure living in London, UK; Hull York Medical School, University of Hull, Cottingham Road, Kingston upon Hull HU6 7RX, UK; School of Cardiovascular & Metabolic Health, University of Glasgow, University Avenue, Glasgow G12 8QQ, UK; Hull University Teaching Hospitals NHS Trust, Castle Hill Hospital, Castle Road, Cottingham, Kingston upon Hull HU16 5JQ, UK; Hull York Medical School, University of Hull, Cottingham Road, Kingston upon Hull HU6 7RX, UK

**Keywords:** Heart failure, Sodium chloride, Diuretics, Diuresis, NaCl

## Abstract

**Aims:**

Intravenous (i.v.) hypertonic saline alongside i.v. loop diuretics is sometimes used to enhance diuresis in people hospitalized with heart failure (HF) but is challenging to administer. Oral sodium chloride (NaCl) supplements might be a practical alternative, but little is known about their effects in patients with HF. We performed a rapid review of the relevant evidence.

**Methods and results:**

A rapid systematic review was registered (PROSPERO: CRD420250618965) and reported following Preferred Reporting Items for Systematic Reviews and Meta-analyses guidelines. Medline and Cochrane Central Register of Controlled Trials databases were searched for studies involving adults with HF administered with oral NaCl. Randomized and observational studies were included. Studies of oral NaCl restriction or i.v. hypertonic NaCl were excluded. All available data were extracted. Risk of bias was evaluated using Risk of Bias 2 and Risk Of Bias In Nonrandomized Studies of Interventions tools. From an initial 335 records, five studies involving 139 patients were included. Oral NaCl did not affect weight or urine volume but were associated with higher serum and urinary sodium concentrations. Some studies reported that NaCl was associated with smaller diuretic-induced increases in serum urea and creatinine, lower haematocrit, higher plasma volume, and less neurohormonal activation compared to normal NaCl intake. Clinical outcomes, including hospital length of stay and mortality, were unaffected. The quality of evidence was limited by small sample sizes and methodological heterogeneity.

**Conclusion:**

For patients with HF treated with loop diuretics, higher oral NaCl intake may increase serum and urine sodium concentration, improve renal function, and reduce neurohormonal activation. There is insufficient evidence to support oral NaCl as an adjunct to diuretic treatment. More research is needed.

## Introduction

Severe congestion due to heart failure (HF) is a common reason for hospitalization in people aged more than 65 years.^1–3^ The cornerstone of treatment for severe congestion is intravenous (i.v.) administration of a loop diuretic.^4^ However, the side effects of loop diuretics, such as neurohormonal activation,^5^ renal dysfunction,^6^ and electrolyte abnormalities,^7,8^ are common and may impair diuretic efficacy.^9^

Combining loop diuretics with adjunctive treatments might reduce the dose of loop diuretic needed to induce an adequate diuresis. Hyponatraemia and hypochloraemia are common in people with advanced HF receiving high doses of i.v. diuretic and may contribute to diuretic resistance.^10^ Sodium chloride (NaCl) supplements might enhance the action of loop diuretics by increasing the availability of sodium and chloride in the nephron,^11^ by increasing renal blood flow,^12^ and by reducing neurohormonal activation.^13^

Several small studies suggest that small-volume i.v. hypertonic saline (HS) given alongside high-dose i.v. loop diuretic causes a greater diuresis, shortens hospital stay, and reduces HF re-hospitalization rates compared to i.v. loop diuretics alone.^14^ However, HS requires central venous access and is logistically difficult to administer safely.^15^ Oral NaCl may be a more practical alternative, but the effects of oral NaCl supplements in patients with HF are unknown.

We performed a rapid review of oral NaCl supplementation (greater than usual recommended daily intake) in patients with symptomatic HF to collate and analyse the available data and assess whether sufficient data exist to consider oral NaCl as a potential adjunctive diuretic treatment in patients with HF. We did not include trials of dietary sodium restriction in this review.

## Methods

This rapid review was designed and conducted in accordance with guidance for rapid reviews by Haby and Garrity,^16,17^ reported in accordance with the Preferred Reporting Items for Systematic Reviews and Meta-analyses (PRISMA) guidelines,^18^ and was registered with PROSPERO (CRD420250618965).^19^

### Study eligibility

We included all primary research studies, randomized or observational, published either in peer-reviewed journals or as abstracts or conference proceedings. Studies that involved adult patients with HF who were given oral NaCl supplementation either as tablets or as part of their diet were included. Studies using i.v. NaCl, or those that assessed NaCl restriction compared to normal intake, were excluded as these have been extensively assessed in systematic reviews and meta-analyses.^14,20–27^ Review articles, non-English language studies, and those not including our patient population or intervention were excluded. No restrictions were made as to patient setting and diuretic dose.

### Search strategy and data extraction

We searched Medline All via OVID and Cochrane Central Register of Controlled Trials (CENTRAL) and the Cochrane Database of Systematic Reviews via The Cochrane Library from inception to 21 February 2025. The search strategy was designed, tested, and peer reviewed within the team, which included an information specialist (S.G.). We examined the indexing of known relevant studies and included historical terms for HF to create a final search combining terms for oral NaCl AND HF. Full search strategies for all databases are shown in Supplementary material online, *Table S1*. References and forward citations of included studies were also assessed for relevant articles. Database results were imported into an Endnote Library where duplicates were removed using a validated method before remaining results were uploaded to Covidence software for title and abstract screening and full-text screening. Data extraction was performed using a bespoke form in Microsoft Excel®. Screening, selection of studies, and extraction were performed by two independent reviewers (D.A. and O.A.). All study selection, and 50% of data extraction, was checked by a senior reviewer (J.J.C.).

Data were extracted on all available baseline characteristics and for the following outcomes relating to diuretic efficacy: weight loss, urine volume, net fluid balance, diuretic dose, and signs of venous congestion on examination. Other data extracted included, but were not limited to, the difference between groups or change from baseline in symptoms of HF; serum concentrations of sodium, chloride, bicarbonate, urea, creatinine, glomerular filtration rate, haematocrit, and natriuretic peptide; urinary electrolyte concentrations; haemodynamic measures (if available); and outcome.

### Quality and risk of bias assessment

The quality of included studies was assessed by two independent reviewers (D.A. and O.A.) using the Cochrane Risk of Bias 2 (ROB-2)^28^ and the Risk Of Bias In Nonrandomized Studies of Interventions (ROBINS-I)^29^ tools for randomized and nonrandomized studies, respectively. Disagreements in the risk of biases assessments were resolved through discussion with a senior reviewer (J.J.C.).

### Statistical analysis

Data are presented as they were in the original published articles. Due to substantial heterogeneity amongst the included studies, a formal meta-analysis was not feasible, and therefore, we adopted a narrative approach. We considered reports of differences between trial arms with *P* < 0.05 as indicators of effect. N-terminal pro-B-type natriuretic peptide (NT-proBNP) concentrations were converted from pmol/L to ng/L by multiplying by 8.5,^30^ and serum creatinine concentrations were converted from mg/dL to µmol/L by multiplying by 88.4.^31^

## Results

### Study selection and baseline characteristics

**Figure 1:**
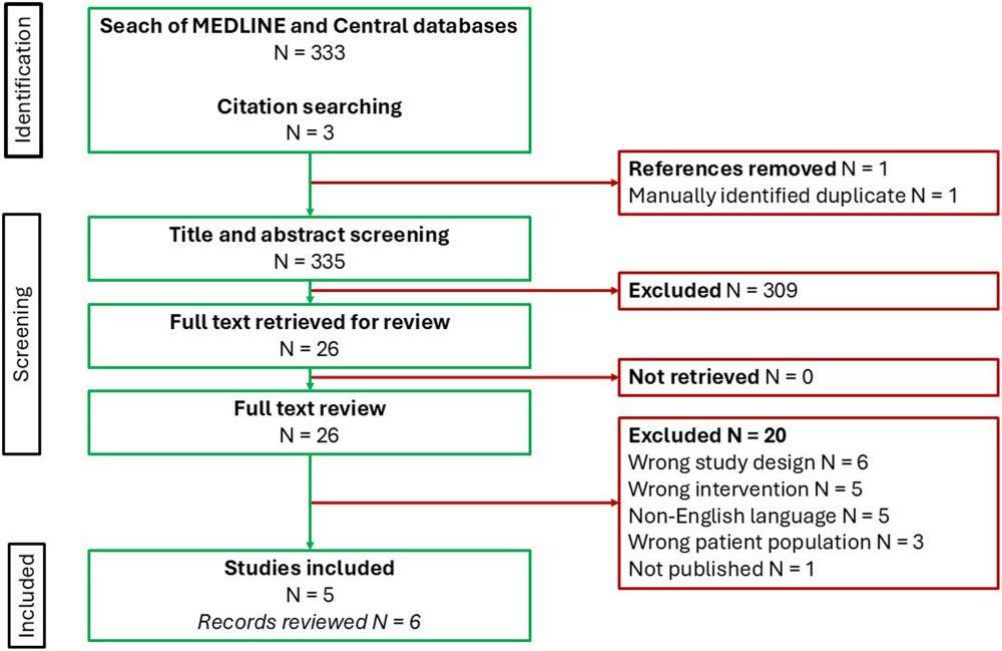
Preferred Reporting Items for Systematic Reviews and Meta-analyses flow diagram.

Of the 334 studies identified, 308 were excluded during title and abstract screening. Of the 26 studies that underwent full-text review, six articles (representing five studies) met our inclusion criteria (one case series, two observational studies, one randomized crossover trial, and one randomized placebo-controlled trial). The studies involved 139 patients with HF (*Figure 1*; *Table 1*).^32–37^

**Table 1:** Study characteristics

Study (year)	Study design	*n*	HF population (% or mean/median values)	Age (years)	Women	NYHA	LVEF	Main exclusion criteria	Type and dose of diuretic	Other HF treatment	Water restriction	Form and dose of NaCl	Compared to	Duration (days)
Waldman (1953)^32^	Case series	4	Chronic congestive	NR	NR	NR	NR	NR	IM mercurial diuretic—2 cc	Digitalis Low salt diet	Not reported	Diet 4 g/d <∼70 mmol/d)	Diet <2 g/d; <∼34 mmol/d	1
Dubiel (1972)^33^	Prospective, observational	23	Outpatients	38	79%	I–II	NR	NR	7 patients received 80 mg of furosemide. Unclear route given	Not reported	Not reported	Tablets	Healthy controls (*n* = 16)	8
												∼7 g/d		
												∼112 mmol/d		
Volpe (1997)^34^	Prospective observational	10	Outpatients	51	20%	I	30%	DM, IHD, hypertension, AF, serum creatinine >104 µmol/L); signs of congestion; recent HF hospitalization;	None—no HF treatment was permitted as per study protocol	None	∼1600 mL/d	Tablets	Diet	8
												∼9 g/d	∼6 g/d	
										Re-studied (*n* = 6) after 6 weeks on enalapril 5 mg/d		150 mmol/d added to control diet	100 mmol/d	
Damgaard (2006)^35^	RCT crossover, open-label	12	Outpatients	57	0	II–III	26%	Recent HFH; AF; ‘abnormal’ blood glucose, serum creatinine, or spirometry	83% taking LD. Dose not reported	100% on ACEI/ARB; 58% on βB; 33% on MRA	Free fluid intake	Diet	Diet	7
												∼15 g/d	4 g/d	
												250 mmol/d	70 mmol/d	
Damgaard (2007)^36,a^	RCT, crossover, open-label	12	Outpatients;	57	0%	II–III	26%	Unable to comply with diet	83% taking loop diuretic. Dose not reported	100% on ACEI/ARB; 100% on ϐB; 33% on MRA	Free fluid intake	Diet	Diet	*7*
												∼15 g/d	4 g/d	
												250 mmol/d	70 mmol/d	
Montgomery (2023)^37^	RCT, Blinded, placebo controlled	65	Hospitalized Median NT-proBNP 4040 ng/L)	70	37%	III–IV	45%	RRT or eGFR <15; Serum sodium <120 or >145 mmol/L; SBP >180 mmHg; anticipated to spend <72 h in hospital.	100% on LD (mean dose 405–460 mg/d)	ACEi/ARNi ∼ 30% ϐB ∼ 70%	At discretion of treating team	Tablets	Diet	4
												6 g/d	2 g/d	
										MRA ∼ 50%		102 mmol/d added to control diet	34 mmol/d	
										SGLT2i ∼ 23%				

^a^Not explicitly stated in the publication, but we suspect these data are the same as the Damgaard (2006) investigation.

HF, heart failure; NYHA, New York Heart Association; RCT, randomized controlled trial; LVEF, left ventricular ejection fraction; ACEI, angiotensin-converting enzyme inhibitor; NT-proBNP, N-terminal pro-B-type natriuretic peptide; DM, diabetes mellitus; IHD, ischaemic heart disease; AF, atrial fibrillation; RRT, renal replacement therapy; HFH, heart failure hospitalization; eGFR, estimated glomerular filtration rate; SBP, systolic blood pressure; IM, intramuscular; LD, loop diuretic; ARB, angiotensin receptor blocker; ϐB, beta-blocker; MRA, mineralocorticoid receptor antagonist; ARNI, angiotensin receptor–neprilysin inhibitor; SGLT2I, sodium–glucose cotransporter 2 inhibitor; g, gram; d, day.

**Figure 2:**
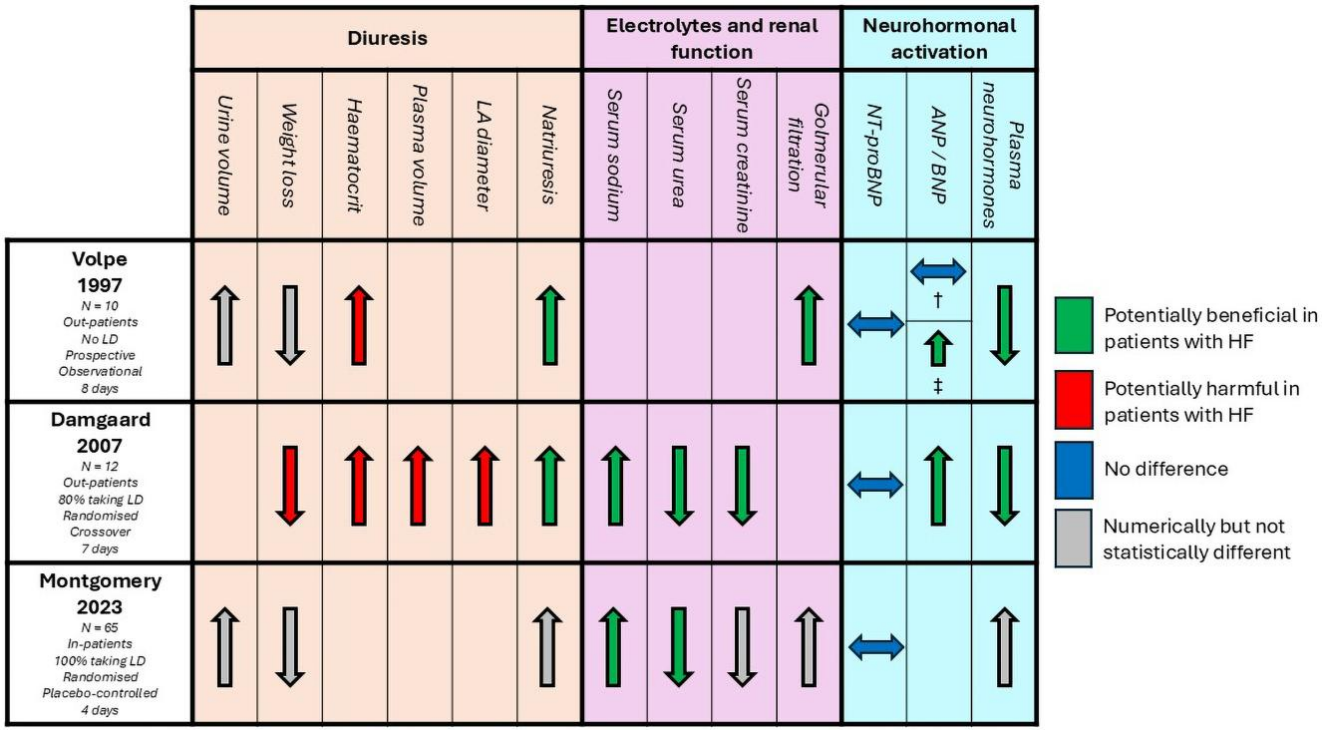
Summary of effects of sodium chloride supplementation in patients with heart failure. Caption: †, in all patients on no HF therapies (*N* = 10); ‡, in a subgroup of patients who repeated the study protocol while taking enalapril 5 mg per day (*N* = 6). LA, left atrial; NT-proBNP, N-terminal pro-B-type natriuretic peptide; LD, loop diuretic; ANP, atrial natriuretic peptide; BNP, B-type natriuretic peptide; HF, heart failure.

There was substantial heterogeneity in the populations studied, the dose and form (tablets or dietary) of oral NaCl given, the duration of the intervention, the control groups or comparisons made, and the outcomes measured. Accordingly, a meta-analysis was not possible (*Tables 1*; Supplementary material online, *Table S2*; *Figure 2*). The nonrandomized studies had either serious or critical risks of bias and were of lower quality; the two randomized trials had a low risk of bias (see Supplementary material online, *Table S3*).

#### Randomized trials

There were two relevant randomized trials (*Table 1*; Supplementary material online, *Table S2*). The first one (Damgaard) was a crossover trial including 12 outpatients with HF and was randomized to either a high- (250 mmol/day) or low-salt (70 mmol/day) diet for 7 days with no interim ‘washout’ periods.^35,36^ However, only 10 of the 12 patients were taking loop diuretics (dose not specified).

The second (Montgomery) was a placebo-controlled trial of 65 patients hospitalized for HF who were randomized to 102 mmol/day NaCl given as Slow Sodium® tablets or matching placebo for 4 days (Montgomery 2023).^37^ All patients in this study were treated with i.v. loop diuretics at a mean dose of 460 mg per day.

### Effect of oral sodium chloride on measures of diuresis

**Figure 3:**
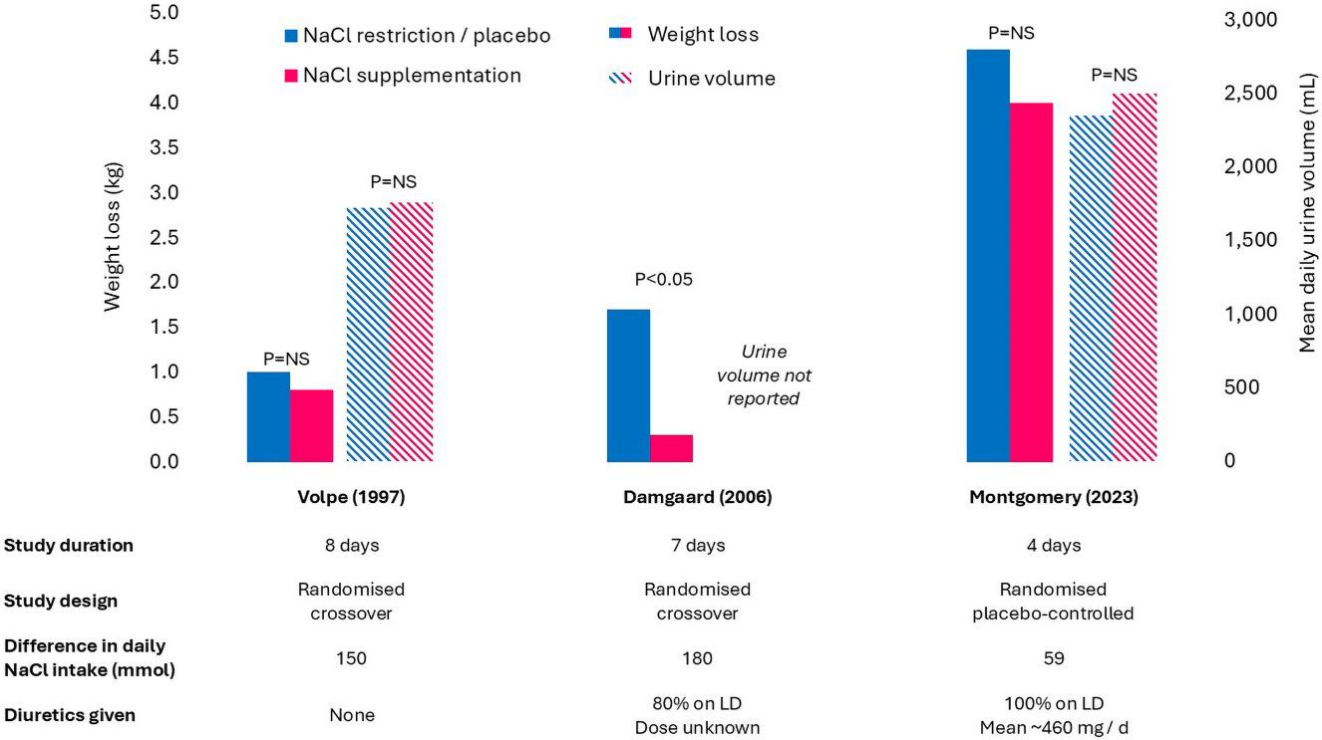
Weight loss and urine volume. NS, not significant; LD, loop diuretic.

The Damgaard trial reported a higher body weight after 7 days on a high salt compared to low-salt diet (90 vs. 92 kg; *P* < 0.005; *Table 2*; *Figure 3*).^35^ Montgomery reported no difference in weight with or without NaCl supplementation.

**Table 2:** Effect of oral sodium chloride supplementation on measures of diuresis

Measure of diuresis	Study (year)	Daily NaCl: intervention (mmol)	Daily NaCl: control (mmol)	Duration of study (days)	Total NaCl: intervention (mmol)	Total NaCl: control (mmol)	Diuretic given	Intervention	Control	Effect of NaCl vs. control	*P*
Urine volume (mL)	Volpe (1997)^34,a^	250	100	8	2000	800	No	14112 ± 1008	13824 ± 1008	–	NS
	Montgomery (2023)^37,a^	103	34	4	412	136	100% i.v. LD; 460 mg/day	10000 ± 4200	9400 ± 4300	–	0.61
Diuretic efficacy (L of urine per 40 mg of furosemide eq)	Montgomery (2023)^37,a^	–	–	–	–	–	–	0.17 (0.06–0.24)	0.14 (0.07–0.21)	–	0.45
Weight loss (kg)	Volpe (1997)^34,b^	250	100	8	2000	800	No	−0.8	−1.0	–	NS
	Damgaard (2006)^35,b^	250	70	7	2000	490	80% taking LD; dose NR	−0.3	−1.7	↓	<0.005
	Montgomery (2023)^37,a^	103	34	4	412	136	100% i.v. LD; 460 mg/day	−4.0 ± 4.3	−4.6 ± 4.2	–	0.57
Body weight at end of study (kg)	Volpe (1997)^34,a^	250	100	8	2000	800	No	66.2 ± 2.2	66.0 ± 2.2	–	NS
	Damgaard (2006)^35,a^	250	70	7	2000	490	80% taking LD; dose NR	92 ± 4	90 ± 4	↑	<0.005
	Montgomery (2023)^37,b^	103	34	4	412	136	100% i.v. LD; 460 mg/day	96.0	94.4	–	NS
Fractional excretion of H_2_O (%)	Volpe (1997)^34,c^	250	100	8	2000	800	No	0.05	0.07	↓	<0.05
Haematocrit (%)	Volpe (1997)^34^	250	100	8	2000	800	No	0.385 ± 0.007	0.416 ± 0.008	↓	<0.05
	Damgaard (2006)^35,a^	250	70	7	2000	490	80% taking LD; dose NR	0.420 ± 0.001	0.440	↓	<0.05
Plasma volume—seated (mL)	Damgaard (2006)^35,a^	250	70	7	2000	490	80% taking LD; dose NR	3608 ± 215	3023 ± 268	↑	<0.05
Plasma volume—supine (mL)	Damgaard (2006)^35,a^	–	–	–	–	–	–	3608 ± 215	3365 ± 246	↑	<0.05
LA diameter (mm)	Damgaard (2006)^35,a^	–	–	–	–	–	–	40 ± 1	38 ± 2	↑	<0.05

^a^Data taken from text or tables.

^b^Data calculated from data available in text and tables.

^c^Data derived from figures.

Urine volume was not measured in the Damgaard trial. In the Montgomery trial, urine volume tended to be higher with oral NaCl supplementation compared to no supplementation, but the difference was small, not likely to be clinically relevant, and not statistically significant (*Figure 3*).^34,37^

In the Damgaard trial, haematocrit was lower, and left atrial diameter and plasma volume were greater, in patients at the end of the high NaCl dietary period (*Table 2*).^36^

### Effect of oral sodium chloride supplementation on urinary electrolytes

Natriuresis was measured in both trials as either as 24-h urinary sodium or spot urine concentration of the first void urine. Natriuresis was significantly greater after NaCl supplementation in Damgaard and numerically greater in Montgomery, but *P* values were not reported (*Table 3*).^35,37^

**Table 3:** Natriuresis

Measure of urinary electrolytes	Study (year)	Daily NaCl: intervention (mmol)	Daily NaCl: control (mmol)	Total NaCl: intervention (mmol)	Total NaCl: control (mmol)	Diuretic given	Intervention	Control	Effect of NaCl vs. control	*P*
Mean urinary NaCl (g)	Waldman (1953)^32,a^	68	17	68	17	2 cc mercurial diuretic	9.2 ± 1.9	6.8 ± 1.3	–	NR
24-h natriuresis (mmol)	Volpe (1997)^34,b^	250	100	2000	800	No	300	100	↑	<0.01
	Damgaard (2006)^35,b^	250	70	2000	490	80% taking LD; dose NR	200	60	↑	<0.05
24-h natriuresis on Day 5 (mmol)	Damgaard (2007)^36,a^	250	70	2000	490	80% taking LD; dose NR	218 ± 31	67 ± 15	↑	NR
24-h natriuresis on Day 6 (mmol)	Damgaard (2007)^36,a^	–	–	–	–	–	209 ± 25	64 ± 15	↑	NR
24-h natriuresis on Day 7 (mmol)	Damgaard (2007)^36,a^	–	–	–	–	–	201 ± 17	60 ± 16	↑	NR
Change in spot urine sodium concentration from baseline (mmol/L)	Montgomery (2023)^37,a^	103	34	412	136	100% i.v. LD; 460 mg/day	−14 ± 39	−33 ± 40	–	0.11
Spot urine concentration Day 1 (mmol/L)	Montgomery (2023)^37,a^	–	–	–	–	–	77 ± 25	77 ± 29	–	NR
Spot urine concentration Day 2 (mmol/L)	Montgomery (2023)^37,a^	–	–	–	–	–	72 ± 51	62 ± 28	–	NR
Spot urine concentration Day 3 (mmol/L)	Montgomery (2023)^37,a^	–	–	–	–	–	68 ± 25	66 ± 29	–	NR
Spot urine concentration Day 4 (mmol/L)	Montgomery (2023)^37,a^	–	–	—	–	–	67 ± 27	46 ± 25	–	NR

^a^Data taken from text or tables.

^b^Data derived from figures.

In the Montgomery trial, fractional excretion of sodium (FENa^+^) tended to be greater with supplementation, but the difference was not statistically significant.^35^ In the same trial, NaCl supplements were not associated with a difference in urine urea, creatinine, or osmolality, but they were associated with greater urinary chloride loss (see Supplementary material online, *Table S4*).^35^

### Effect of oral sodium chloride supplementation on serum electrolytes and renal function

In both trials, NaCl supplementation was associated with higher serum sodium concentration and a modest but statistically significantly lower serum urea concentration and lesser increase in serum urea during treatment with diuretic (*Table 4*).^35,37^

**Table 4:** Serum electrolytes and renal function: measured at the end of the study period or change from baseline

Serum electrolyte measurement	Study (year)	Daily NaCl: intervention (mmol)	Daily NaCl: control (mmol)	Duration of study (days)	Total NaCl: intervention (mmol)	Total NaCl: control (mmol)	Diuretic given	Intervention	Control	Effect of NaCl vs. control	*P*
Serum sodium (mmol/L)	Damgaard (2006)^35,a^	250	70	7	2000	490	80% taking LD; dose NR	138 ± 0.5	135 ± 0.3	↑	<0.05
	Montgomery (2023)^37,b^	103	34	4	412	136	100% i.v. LD; 460 mg/day	140	137	–	–
Change in serum sodium (mmol/L)	Montgomery (2023)^37,a^	103	34	4	412	136	100% i.v. LD; 460 mg/day	0.0 ± 3.3	−2.6 ± 2.7	↑	<0.001
Serum chloride (mmol/L)	Montgomery (2023)^37,b^	–	–	–	–	–	–	95	95	–	–
Change in serum chloride (mmol/L)	Montgomery (2023)^37,a^	–	–	–	–	–	–	−1 (−4–1)	−3 (−5 to −1)	–	0.19
Serum bicarbonate (mmol/L)	Montgomery (2023)^37,b^	–	–	–	–	–	–	28.5	27.0	–	–
Change in serum bicarbonate (mmol/L)	Montgomery (2023)^37^	–	–	–	–	–	–	1.5 (−2.0–3.8)	0 (−3.5–4.0)	–	0.44
Serum osmolality (mOsm/kg H_2_O)	Volpe (1997)^34^	250	100	8	2000	800	No	288 ± 3	286 ± 2	–	NS
Serum urea (mmol/L)	Damgaard (2006)^35,a^	250	70	7	2000	490	80% taking LD; dose NR	5.9 ± 0.5	8.4 ± 1.4	↓	<0.05
Change in serum urea (mmol/L)	Montgomery (2023)^37,a^	103	34	4	412	136	100% i.v. LD; 460 mg/day	3.1 ± 13	11 ± 15	↓	0.03
Serum creatinine (μmol/L)	Damgaard (2006)^35,a^	250	70	7	2000	490	80% taking LD; dose NR	102 ± 5	110 ± 6	↓	<0.05
	Montgomery (2023)^37,b^	103	34	4	412	136	100% i.v. LD; 460 mg/day	180	155	–	–
Change in serum creatinine (μmol/L)	Montgomery (2023)^37,a^	103	34	4	412	136	100% i.v. LD; 460 mg/day	4 ± 34	13 ± 39	–	0.30
eGFR (mL/min/1.73 m^2^)	Montgomery (2023)^37,b^	–	–	–	–	–	–	39	39	–	–
Change in eGFR (mL/min/1.73 m^2^)	Montgomery (2023)^37,a^	–	–	–	–	–	–	1.7 (−4.6–4.1)	−2.8 (−7.5–3.1)	–	0.25
GFR (mL/min)	Volpe (1997)^34,c^	250	100	8	2000	800	No	130	110	**↑**	<0.01

^a^Data taken from text or tables.

^b^Data calculated from data available in text and tables.

^c^Data derived from figures.

### Effect of oral sodium chloride supplementation on haemodynamic and neurohormonal measurements

In the Damgaard trial, NaCl supplementation was associated with greater cardiac index and renal blood flow, and lower renovascular and total peripheral vascular resistance, but had no effect mean arterial pressure (see Supplementary material online, *Table S5*).^34,35^ In the same trial, serum noradrenaline and plasma angiotensin II concentrations were lower with NaCl supplementation.^35^ However, in the Montgomery trial, there was no statistically significant difference in the change in aldosterone concentrations (the only HF-associated neurohormone measured in the trial) between baseline and Day 4 (see Supplementary material online, *Table S6*).^37^

In both trials, there was no difference in NT-proBNP or change in NT-proBNP from baseline with NaCl supplements.^36,37^ However, the Damgaard trial reported a significant increase in B-type natriuretic peptide (BNP) concentration (69% increase, baseline data not reported) after NaCl supplementation compared to restriction (see Supplementary material online, *Table S6*).^36^

### Effect of oral sodium chloride supplementation on outcome

Only Montgomery reported clinical outcomes and *P* values were not reported. However, oral NaCl supplementation appeared to have no impact on length of stay in the hospital, nor all-cause hospitalization or mortality after discharge (see Supplementary material online, *Table S7*).^35^

#### Observational studies

Three observational studies were identified, all published before the year 2000, involving 37 patients with HF: one case series from 1953 of four outpatients with HF (Waldman 1953);^32^ one prospective, case–control, observational study of 23 outpatients with HF who received 900 mmol of NaCl in total in divided doses over 8 days compared to 16 healthy controls (Dubiel 1972);^33^ and one prospective observational study of 10 outpatients with HF who were taking no other medical therapy and who received a low-sodium diet for 6 days (control period) followed by 150 mmol NaCl in divided doses plus a high-sodium diet (250 mmol/day in total) for 8 days (Volpe 1997).^34^ In Volpe *et al.*,^34^ the protocol was repeated 6 weeks later in six patients who were also taking enalapril.

- Natriuresis, measured as either mean urine sodium content in grams (Waldman 1953), 24-h urine sodium content in millimole (Volpe 1997), or filtered sodium load in mmol/min (Volpe 1997), was greater with supplementation.^32,34^
- In the Volpe study:
  - Sodium chloride supplements had no impact on urine volume, weight loss, or overall body weight. However, fractional excretion of water was lower, and haematocrit was higher with NaCl supplementation.^34^
  - Fractional excretion of sodium was lower with supplementation, but in the subgroup of patients who repeated the study protocol while taking enalapril (but no diuretic), FENa^+^ was *greater* with supplementation (*P* < 0.05).^34^
  - Renal blood flow was higher, and renovascular resistance was lower, with NaCl supplements. As a consequence, renal function, measured as glomerular filtration (mL/min), was also greater with NaCl supplements.^34^
  - Plasma renin activity and plasma aldosterone concentrations were lower with NaCl supplements.^34^
  - There was no difference in plasma atrial natriuretic peptide (ANP) concentrations in patients prior to receiving enalapril, but there was a significant increase in ANP during the NaCl supplement after taking enalapril (see Supplementary material online, *Table S7*).^34^
- No hospitalization or mortality data were reported in any observational study.

## Discussion

The data presented are from single-centre studies, involving small numbers of patients, predominantly performed in the era before quadruple therapy for HF; they are thus weak and very limited. There is insufficient evidence to say whether NaCl supplementation has any diuretic effect in patients with HF. There are some signals that suggest not only potential mechanisms of benefit but also potential safety concerns.

### Is sodium chloride supplementation beneficial in patients with heart failure?

#### Renal function

We found weak evidence from one observational study and two randomized trials that oral NaCl supplementation may be associated with better renal function in stable outpatients^34,35^ and a lower rise in markers of worsening renal function in patients receiving high-dose i.v. loop diuretics during hospitalization.^37^ Serum creatinine and the increase in serum creatinine from baseline were also numerically lower, but the difference was not statistically significant.^35,37^

Worsening renal function is a common reason for stopping (or reducing the dose of) loop diuretic treatment in patients with HF and venous congestion, both in and out of hospital.^38,39^ If oral NaCl reduces the risk of worsening renal function during diuresis, it may enable longer term treatment with higher doses of loop diuretic.

#### Natriuresis

Perhaps unsurprisingly, oral NaCl supplementation was associated with greater serum and urine sodium concentrations. Increased natriuresis is associated with increased diuresis,^40^ and it is common dogma that the amount of sodium in the urine is a guide to the amount of diuresis to follow.^41^ Using natriuresis to guide diuretic treatment is recommended by the European Society of Cardiology HF guidelines and, as a strategy, has been subject to several clinical trials.^42–44^ While this approach may be flawed,^45^ it is almost certainly true that the mechanism by which loop diuretics work is by increasing natriuresis. Theoretically, at least, any treatment that increases natriuresis may also, concurrently, increase diuresis. However, the theory is not borne out in the available data: we found only small and statistically nonsignificant increases in urine volume with oral NaCl supplementation, despite large and statistically significant differences in urine sodium content.

#### Neurohormonal activation

The available data also suggest that oral NaCl supplementation may be associated with reduced activation of the renin–angiotensin–aldosterone system (RAAS) and sympathetic nervous system (SNS). These data mirror findings in animal HF models.^46^ Higher serum and urine sodium and chloride concentrations are likely to supress renin secretion from the macula densa, thus reducing serum concentrations of other RAAS hormones, and subsequent downstream activation of the SNS.^47^

Activation of the RAAS is thought to be a key driver of congestion in HF, but some data suggest that plasma concentrations of RAAS hormones only increase above the normal range *after* initiation of diuretic treatment.^5^ One observational study and one randomized trial found statistically significant reductions in serum RAAS and SNS hormone concentrations. The observational study involved patients who were not taking diuretic therapy;^34^ and in the randomized trial, 80% of patients (*n* = 10) were taking loop diuretics.^35^

In one randomized trial in patients hospitalized with venous congestion, all of whom were taking loop diuretics, aldosterone concentration increased from baseline in patients receiving oral NaCl and decreased in the placebo arm, but the difference was not statistically significant.^35^ Baseline data were not reported, so it is unclear if these changes are just regression to the mean.

### Is oral sodium chloride supplementation harmful in patients with heart failure?

#### Plasma volume

The dogma of salt restriction in patients with HF is rooted in the belief that salt (and, therefore, water) retention drives worsening congestion and outcomes. Increased dietary salt increases the amount of salt available for absorption and, in turn, increases the amount of renal water absorption. We have identified some data to support this hypothesis, but it is unclear whether the changes are clinically meaningful.

One trial reported a small but statistically significant increase in plasma volume, left atrial diameter, and cardiac index;^35^ one observational study and one randomized trial reported a significantly lower haematocrit and a significant increase in serum natriuretic peptide concentrations with supplementation.^34,36^ Each may reflect increased renal water retention due to increased NaCl within the renal tubule.

Sodium chloride supplementation was also associated with numerically less weight loss than no supplementation.^34,35,37^ Two randomized trials also reported lower serum urea^35^ or a lesser increase in serum urea with supplementation while also reporting no difference in serum creatinine or glomerular filtration rate:^37^ this, too, may reflect haemodilution. It is plausible that any diuretic effect of oral NaCl supplementation is cancelled out by increased water retention, leading to a neutral overall effect on diuresis. Increased oral salt intake leads to water retention in order to maintain the extracellular concentrations of sodium and chloride. Increased plasma volume would, in turn, be expected to increase secretion of natriuretic peptides and reduce activation of systems designed to retain sodium, including renin, angiotensin II, aldosterone, and noradrenaline. Consequently, the brake on salt retention is relieved, allowing a greater diuresis, perhaps especially amongst hyponatraemic patients.

It is important to note that the symptoms of HF are due to organ and tissue congestion, not increased plasma volume. While the latter often precedes the former during decompensation, the data available suggest that short-term increases in plasma volume during treatment with loop diuretic may improve renal perfusion and preserve renal function.

#### Supplementation or restriction?

One weakness of the data is the lack of certainty regarding the quantity of sodium given. The majority of dietary sodium comes from table salt, which is ∼50% sodium.^48^ One study and one trial reported sodium restriction to 70–100 mmol of sodium per day as the control group (Volpe 1997; Damgaard 2006). However, to provide 70–100 mmol of sodium per day in the diet, 4–6 g of sodium chloride per day is needed. Given that some HF guidelines recommend that dietary salt intake should be restricted to no more than 2 g per day,^49^ or not to exceed 5 g per day,^42^ the patients were not, in fact, on a ‘low-sodium diet’ by modern standards. However, in both the study and the trial, the intervention arm received 250 mmol of sodium per day (either as part of the diet or as NaCl tablets), which equates to around 15 g of table salt; which is, undoubtedly, high sodium-chloride intake, and thus an appropriate intervention for the purposes of this review.

We excluded three other studies (Paterna 2008; Parinello 2009; Paterna 2009)^50–52^ because they were reported as being studies of sodium restriction. Each compared daily dietary sodium intake of 80 mmol to an intake of 120 mmol, which is 1.8 compared to 2.8 g of sodium per day. This quantity of sodium as NaCl is 5 vs. 7 g of NaCl per day. Each of these studies thus may have included a group that involved sodium chloride supplementation (greater than recommended oral NaCl intake). Our calculations are correct based on the molar mass of sodium, but it is possible that the amounts were misreported. Due to uncertainty, and the inclusion of these studies in previous systematic reviews of sodium restriction,^25^ we did not include them in the present analysis.

Although the findings of our review are limited, they align with findings of previous reviews of sodium restriction.^25,26^ Patients who receive more oral NaCl (as part of a normal diet compared to sodium restriction) have lower serum concentrations of neurohormones associated with HF progression,^50–55^ greater blood pressure and, possibly, renal perfusion,^52,53,55^ better renal function,^52,53^ and greater natriuresis and urine output.^52,54^

Some individual trials of sodium restriction have suggested a trend towards improved symptoms and outcome with restriction in patients with HF,^56–58^ while others have indicated possible detriment to quality of life.^59,60^ Although the overall effect of sodium restriction on symptoms and outcome in patients with HF across multiple systematic reviews, meta-analyses, and one large, multinational randomized controlled trial (RCT) appears to be neutral,^22–27,61^ one analysis suggested a greater risk of adverse outcome with salt restriction compared to normal sodium diet in hospitalized patients receiving i.v. loop diuretic.^25^ It remains to be seen whether sodium *supplementation* might improve diuresis and, therefore, potentially, symptoms, quality of life, and outcomes in patients with severe venous congestion requiring high-dose diuretic treatment.

#### Limitations

To our knowledge, this is the first systematic review of oral NaCl supplementation in patients with HF. We excluded studies of NaCl restriction as these have already been subject to meta-analyses and would have clouded our investigation of the effects of greater-than-normal levels of sodium intake.

This review uses rapid review methods, including searching a limited number of databases, which increases the possibility of missing relevant studies. However, a broad search strategy and a supplementary citation search limit the likelihood of missing appropriate papers.

The studies identified were highly heterogenous in population, intervention, study design, and outcome measured and involved only a small number of patients with only one trial taking place after 2010. The findings are therefore limited. However, taken together with data from sodium restriction trials, there are some signals that support further investigation of oral NaCl supplementation to protect against worsening renal function and neurohormonal activation and, possibly, to increase diuretics in patients with HF who are receiving diuretic treatment. By the same token, the negative effects of sodium chloride supplementation may outweigh the potential beneficial effects in stable outpatients, regardless of HF phenotype, who are not taking high-dose loop diuretic.

## Conclusions

Oral NaCl supplementation in patients with HF may be associated with better renal function and less neurohormonal activation, but also greater water retention, which may negate any potential diuretic effect. More work is needed to establish the efficacy of oral NaCl as an adjunct to diuretic treatment in patients with HF.

## Supplementary Material

oeag017_Supplementary_Data

## Data Availability

Data reported here are from other published sources but our synthesized dataset would be made available upon reasonable request to the corresponding author.
