# Supplementary material for: A rapid review and narrative synthesis of the evidence for oral sodium chloride supplements in the management of heart failure
Source: Eur Heart J Open. 2026 Feb 6;6(2):oeag017. doi: 10.1093/ehjopen/oeag017 (PMC12978528; doi:10.1093/ehjopen/oeag017)
Supplement: oeag017_Supplementary_Data [file oeag017_supplementary_data.docx]

**Supplementary Table 1**

| **#** | **Query** |
| --- | --- |
| 1 | exp Heart Failure/ |
| 2 | (heart failure or HF).ti,ab,kw,kf. |
| 3 | ((heart or cardiac) adj3 failure).mp. |
| 4 | (congestive adj3 heart).mp. |
| 5 | (circulatory adj3 failure).mp. |
| 6 | (myocardial adj3 insufficiency).mp. |
| 7 | (HFrEF or HFpEF or HFmrEF or HFimpEF).mp. [to capture HF with reduced ejection fraction etc] |
| 8 | or/1-7 [heart failure concept] |
| 9 | Administration, Oral/ |
| 10 | exp Sodium/ |
| 11 | 9 and 10 |
| 12 | Sodium Chloride/ad, de, tu |
| 13 | (((oral* or supplement*) adj2 (sodium or salt* or NaCl)) or slow sodium).mp. |
| 14 | 11 or 12 or 13 |
| 15 | 8 and 14 |
| 16 | (*Sodium Chloride/ and *Heart Failure/) or (sodium chloride and heart failure).ti. |
| 17 | 15 or 16 |

**Supplementary table 2 – Outcomes measured**

| **Study (year)** | **Weight** | **Diuresis** | **Natri-uresis** | **Plasma Voume** | **Comments** |
| --- | --- | --- | --- | --- | --- |
| Waldman (1953) | No | No | Yes | No | Mean 24 hour urinary NaCl output (g) |
| Dubiel (1972) | No | No | Yes | Yes | Cumulative natriuresis during study (mmol); change in plasma volume^b^ |
| Volpe (1997) | Yes | Yes | Yes | No | Heart rate (bpm); systolic BP (mmHg); body weight (kg); urine volume (mL); urine osmolality (mOsm/kg H_2_O); plasma osmolality (mOsm/kg H_2_O); renal blood flow (mL/min); filtered Na^+^ (mmol/min)^c^; fractional excretion of Na^+^ (%)^c^; fractional excretion of H_2_O (%)^c^; renal vascular resistance (mmHg/mL/min) ^c^; 24-hr natriuresis (mmol) ^c^; glomerular filtration rate (mL/min)^c^; plasma renin activity (ng/mL/hr)^c^; plasma aldosterone (pg/mL)^c^; plasma ANP, BNP, and noradrenaline (pg/mL)^d^ |
| Damgaard (2006) | Yes | Yes | Yes | Yes | 24-hr natriuresis (mmol)†; body weight (kg); seated plasma volume (mL)^b^; supine plasma volume (mL)^b^; serum sodium (mmol/L); haematocrit (fraction); creatinine (μmol/L); urea (mmol/L); cardiac index (L/min/m2); mean arterial pressure (mmHg); total peripheral resistance (dyns.s.cm-5) LA diameter (mm); serum noradrenaline (pg/mL); serum adrenaline (ng/mL); serum angiotensin II (pg/mL); serum NT-proBNP (pmol/L) |
| *Damgaard (2007)* |  |  | *Yes* | *Yes* | *24-hr natriuresis on day 5 (mmol); 24-hr natriuresis on day 6 (mmol); 24-hr natriuresis on day 7 (mmol); plasma volume (mL)^b^; BNP ratio (low to high salt)* |
| Montgomery (2023) | Yes | Yes | Yes | Yes | Weight loss (kg); creatinine (mEq/L); change in thirst score; urine output (mL); cumulative furosemide dose (mg); diuretic efficacy (L of urine per 40 mg of furosemide equivalents given); change in Na^+^, Cl^-^, HCO^-^, Urea (all mEq) and eGFR (mL/min/1.73m2) between baseline and day 4; days to discharge; all-cause readmission (%); all-cause death (%).  Other endpoints measured in 97% of study participants: 24 hour Urinalysis measured at 96 hours comprised of natriuresis (mEq/L), urea (mEq/L), chloride (mEq/L), creatinine (mg/dL); fractional excretion of Na^+^ (%); fractional excretion of urea (%); change in natriuresis (mEq/L), urinary chloride (mEq/L), urinary urea (mEq/L), and urinary creatinine (mg/dL) from baseline to 96 hours; change in serum aldosterone (pg/mL) and NT-proBNP (pg/mL) from baseline to 96 hours; spot urine Na^+^ in first void urine measured on days 1-4 (units not given) |

**Legend**

a - Measured on final day of study unless otherwise stated; b – calculated using radio-iodine labelled albumin; c - data not given, estimates taken from figures; d – qualitative data only, no quantitative data given in text, tables, or figures

**Supplementary table 3a - Quality assessment**

|  | **Study design** | **Sequence generation** | **Allocation concealment** | **Blinding of participants and personnel** | **Blinding of outcome assessment** | **Incomplete outcome data** | **Selective reporting** | **Other sources of bias** |
| --- | --- | --- | --- | --- | --- | --- | --- | --- |
| **Waldman (1953)** | Observational | N/A | N/A | High | High | High | Unsure | High |
| **Dubiel (1972)** | Observational | N/A | N/A | High | Low | High | Unsure | High |
| **Volpe**  **(1997)** | Prospective, observational | N/A | N/A | High | Low | Low | Low | Low |
| **Damgaard (2006)** | Randomised, crossover | Low | High | High | Low | Low | Low | Low |
| **Montgomery (2023)** | Randomised, placebo controlled, double blind | Low | Low | Low | Low | Low | Low | Low |

**Legend**

Low – low risk of bias; high – high risk of bias

**Supplemetnary table 3b - ROBINS-I**

|  | **Confounding** | **Classification of intervention** | **Selection of participants** | **Deviations from intended interventions** | **Missing data** | **Measurement of outcome** | **Selective reporting** | **Overall** |
| --- | --- | --- | --- | --- | --- | --- | --- | --- |
| **Waldman (1953)** | Critical | Moderate | Serious | Moderate | Serious | Serious | Serious | **Critical** |
| **Dubiel (1972)** | Moderate | Low | Low | Moderate | Serious | Low | Serious | **Serious** |
| **Volpe (1997)** | Low | Serious | Low | Moderate | Low | Low | Moderate | **Moderate** |

**Supplemetnary table 3c - ROB-2**

|  | **Randomisation** | **Deviations** | **Missing data** | **Measurement of outcome** | **Reported result** | **Overall** |
| --- | --- | --- | --- | --- | --- | --- |
| **Damgaard (2006)** | Low | Some concern | Low | Low | Low | Low |
| **Montgomery (2023)** | Low | Low | Low | Low | Low | Low |

**Supplementary Table 4 – urinary electrolytes**

| **Measure of urinary electrolytes** | **Study (year)** | **Daily NaCl: intervention (mmol)** | **Daily NaCl: control (mmol)** | **Total NaCl: intervention (mmol)** | **Total NaCl: control (mmol)** | **Diuretic given** | **Intervention** | **Control** | **Effect of NaCl vs control** | **P** |
| --- | --- | --- | --- | --- | --- | --- | --- | --- | --- | --- |
| Fractional excretion of sodium (%) | **Volpe**  **(1997)^c^** | 250 | 100 | 2000 | 800 | No | **0.80** | **0.70** | **🡩** | **<0.05** |
|  | Montgomery (2023)^a^ | 103 | 34 | 412 | 136 | 100% IV LD; 460 mg / day | 1.6 (0.2 – 3.3) | 1.1 (0.3 – 3.0) |  | 0.38 |
| Filtered Na^+^ load (mmol/min) | **Volpe**  **(1997)^c^** | 250 | 100 | 2000 | 800 | No | **17.5** | **15.0** | **🡩** | **<0.01** |
| Urinary urea (mmol/L) | Montgomery (2023)^a^ | 103 | 34 | 412 | 136 | 100% IV LD; 460 mg / day | 380 ±220 | 440 ±280 |  | 0.42 |
| Change in urinary urea (mmol/L) | Montgomery (2023)^a^ | - | - | - | - | - | 170 ±240 | 210 ±270 |  | 0.57 |
| Urinary creatinine (μmol/L) | Montgomery (2023)^a^ | - | - | - | - | - | 54 ±37 | 53 ±33 |  | 0.92 |
| Change in urinary creatinine (μmol/L) | Montgomery (2023)^a^ | - | - | - | - | - | 25 ±39 | 16 ±44 |  | 0.37 |
| Urinary chloride (mmol/L) | **Montgomery (2023)^a^** | - | - | - | - | - | **70 ±27** | **50 ±22** | **🡩** | **0.01** |
| Change in urinary chloride (mmol/L) | Montgomery (2023)^a^ | - | - | - | - | - | -16 ±33 | -31 ±36 |  | 0.20 |
| Urine osmolality (mOsm/kg H_2_O) | Volpe  (1997)^a^ | 250 | 100 | 2000 | 800 | No | 105 ±26 | 93 ±17 |  | NS |

**Legend**

a – data taken from text or tables; b – data calculated from data available in text and tables; c – data derived from figures; d – quantitative data not given; e – conversion from pmol/L to ng/L by factor 8.5x;

**Supplementary Table 5 – haemodynamic measures** **recorded at the end of the study period**

| **Haemodynamic measure** | **Study (year)** | **Daily NaCl: intervention (mmol)** | **Daily NaCl: control (mmol)** | **Duration of study (days)** | **Total NaCl: intervention (mmol)** | **Total NaCl: control**  **(mmol)** | **Diuretic given** | **Intervention** | **Control** | **Effect of NaCl vs control** | **P** |
| --- | --- | --- | --- | --- | --- | --- | --- | --- | --- | --- | --- |
| Renal blood flow (mL/min) | **Volpe**  **(1997)^a^** | 250 | 100 | 8 | 2000 | 800 | No | 968 ±71 | 852 ±62 | **↑** | **<0.001** |
| Renal vascular resistance (mmHg/mL/min) | **Volpe**  **(1997)^c^** | - | - | - | - | - | - | 0.100 | 0.125 | **↓** | **<0.05** |
| Cardiac index (L/min) | **Damgaard (2006)^a^** | 250 | 70 | 7 | 2000 | 490 | 80% taking LD; dose NR | 2.22 ±0.11 | 2.08 ±0.13 | **↑** | **<0.05** |
| Total peripheral resistance (dyns.s.cm^-5^) | **Damgaard (2006)^a^** | - | - | - | - | - | - | 1628 ±114 | 1854 ±139 | **↓** | **0.05** |
| Heart rate (bpm) | Volpe  (1997)^a^ | 250 | 100 | 8 | 2000 | 800 | No | 71 ±2 | 70 ±3 | - | NS |
| Systolic blood pressure (mmHg) | Volpe  (1997)^a^ | - | - | - | - | - | - | 115 ±3 | 114 ±6 | - | NS |
| Mean arterial pressure | Damgaard (2006)^a^ | 250 | 70 | 7 | 2000 | 490 | 80% taking LD; dose NR | 80 ±2 | 80 ±2 | - | NS |

**Legend**

All data recorded at the end of the study period (control or intervention) for each study. a – data taken from text or tables; b – data calculated from data available in text and tables; c – data derived from figures; d – quantitative data not given; e – conversion from pmol/L to ng/L by factor 8.5x;

**Supp Table 6 – Measures of neurohormonal activity at the end of the study period**

| **Neurohormonal measure** | **Study (year)** | **Daily NaCl: intervention (mmol)** | **Daily NaCl: control (mmol)** | **Duration of study (days)** | **Total NaCl: intervention (mmol)** | **Total NaCl: control (mmol)** | **Diuretic given** | **Intervention** | **Control** | **Effect of NaCl vs control** | **P** |
| --- | --- | --- | --- | --- | --- | --- | --- | --- | --- | --- | --- |
| NT-proBNP (ng/L) | Damgaard (2006)^a,e^ | - | - | - | - | - | - | 251  (119 – 825) | 242  (119 – 293) | - | NS |
|  | Montgomery (2023)^b^ | 103 | 34 | 4 | 412 | 136 | 100% IV LD; 460 mg / day | 3490 | 2510 | - | NR |
| Change in NT-proBNP from baseline (ng/L) | Montgomery (2023)^a^ | - | - | - | - | - | - | -1050  (-3050 – 207) | -1040  (-1730 – 212) | - | 0.52 |
| ANP (ng/L) | Volpe  (1997)^d^ | 250 | 100 | 8 | 2000 | 800 | No | No difference between high and low salt crossover periods; ANP increased during high salt intake in patients given enalapril | | | |
| BNP (ng/L) | Volpe  (1997)^d^ | - | - | - | - | - | - |  |  |  |  |
|  | Damgaard (2007)^a^ | 250 | 70 | 7 | 2000 | 490 | 80% taking LD; dose NR | BNP increased by 69% (25 – 129%) during high salt crossover period | | | |
| Plasma renin activity (ng/mL/hr) | **Volpe**  **(1997)^c^** | 250 | 100 | 8 | 2000 | 800 | No | **1.5** | **3.0** | **↓** | **<0.05** |
| Aldosterone (pg/mL) | **Volpe**  **(1997)^c^** | - | - | - | - | - | - | **<50** | **125** | **↓** | **<0.05** |
| Change in aldosterone from baseline (pg/mL) | Montgomery (2023)^a^ | 103 | 34 | 4 | 412 | 136 | 100% IV LD; 460 mg / day | 17  (-51 – 123) | -47  (-116 – 85) | - | 0.32 |
| Noradrenaline (pg/mL) | Damgaard (2006) | 250 | 70 | 7 | 2000 | 490 | 80% taking LD; dose NR | **336**  **(247 – 458)** | **503**  **(376 – 673)** | **↓** | **<0.05** |
| Adrenaline (ng/mL) | Damgaard (2006) | - | - | - | - | - | - | 0.04  (0.02 – 0.06) | 0.05  (0.03 – 0.06) | - | NS |
| Angiotensin-II (pg/mL) | Damgaard (2006) | - | - | - | - | - | - | 9.6  (5.2 – 17.9) | 21.0  (12.5 – 35.5) | **↓** | **<0.05** |

**Legend**

a – data taken from text or tables; b – data calculated from data available in text and tables; c – data derived from figures; d – quantitative data not given; e – conversion from pmol/L to ng/L by factor 8.5x;

**Supplementary Table7 - outcome**

| **Endpoint** | **Study (year)** | **Daily dose of NaCl given – intervention (mmol)** | **Daily dose of NaCl given – control (mmol)** | **Duration of study (days)** | **Total NaCl dose in intervention arm (mmol)** | **Total NaCl dose in control arm (mmol)** | **Diuretic given** | **Intervention arm** | **Control arm** | **P** |
| --- | --- | --- | --- | --- | --- | --- | --- | --- | --- | --- |
| Days to discharge | Montgomery (2023) | 103 | 34 | 4 | 412 | 136 | 100% IV LD; 460 mg / day | 8 (6 – 13) | 7 (5 – 14) | NR |
| All-cause readmission (%) | Montgomery (2023) | 103 | 34 | 4 | 412 | 136 | 100% IV LD; 460 mg / day | 21 | 23 | NR |
| All-cause death (%) | Montgomery (2023) | 103 | 34 | 4 | 412 | 136 | 100% IV LD; 460 mg / day | 12 | 10 | NR |

**Legend**

a – data taken from text or tables; b – data calculated from data available in text and tables; c – data derived from figures; d – quantitative data not given; e – conversion from pmol/L to ng/L by factor 8.5x;
